# E-DQN-Based Path Planning Method for Drones in Airsim Simulator under Unknown Environment

**DOI:** 10.3390/biomimetics9040238

**Published:** 2024-04-16

**Authors:** Yixun Chao, Rüdiger Dillmann, Arne Roennau, Zhi Xiong

**Affiliations:** 1 Navigation Research Center, School of Automation Engineering, Nanjing University of Aeronautics and Astronautics, Nanjing 211106, China; 2 FZI Research Center for Information Technology, 76131 Karlsruhe, Germany

**Keywords:** biological inspiration, E-DQN, reinforcement learning, drone, airsim, unreal engine

## Abstract

To improve the rapidity of path planning for drones in unknown environments, a new bio-inspired path planning method using E-DQN (event-based deep *Q*-network), referring to introducing event stream to reinforcement learning network, is proposed. Firstly, event data are collected through an airsim simulator for environmental perception, and an auto-encoder is presented to extract data features and generate event weights. Then, event weights are input into DQN (deep *Q*-network) to choose the action of the next step. Finally, simulation and verification experiments are conducted in a virtual obstacle environment built with an unreal engine and airsim. The experiment results show that the proposed algorithm is adaptable for drones to find the goal in unknown environments and can improve the rapidity of path planning compared with that of commonly used methods.

## 1. Introduction

Due to their small size, low cost, and high maneuverability, drones are widely used in many applications, including target search, disaster rescue, electric power inspection, and so on. Among various capabilities of drones, autonomous path planning plays an important role in guaranteeing the task accomplishment of drones [1,2]. Despite extensive research on motion decision algorithms having been carried out, fast and accurate path planning in complex unknown environments remains challenging for drones.

Traditional path-planning algorithms mainly include RRT (rapidly exploring random tree), artificial potential field, A* algorithm, and so on [3,4,5,6]. Nguyen, T.H. et al., proposed a new path-planning method by inserting a universal pseudo-random number generator into RRT, which improved the convergence and effectiveness of path planning for the mobile robot [7]. Sabudin, E.N. et al., suggested a path potential field-based planning algorithm, which was capable of eliminating the local minima that frequently occurs in the conventional potential field while fulfilling the criterion of path planning and integrates path pruning to shorten the planned path [8]. To optimize path length and path smoothness, Zhang, L. et al., introduced an improved A* algorithm by dividing the distance between adjacent nodes and employing a cubic spline function to smooth the path [9]. For the intelligent unmanned system, the motion environment is not completely known. Certain adaptive and self-learning abilities are needed to adapt to changes in the environment and reach goals quickly. Although the above methods can solve the problems of the local minimum value of traditional path-planning algorithms to some extent, they are not suitable for fast path planning in the large-scale complex flight environments of drones.

With the development of AI (artificial intelligence), many AI-based path-planning algorithms have been conducted, providing inspiration for solving the problems of traditional motion decision-making algorithms. Machmudah, A. et al., addressed the optimization of flight trajectories for a fixed-wing UAV (unmanned aerial vehicle) at a constant altitude by employing a Bezier curve and meta-heuristic optimizations, including PSO (particle swarm optimization), which minimized the path length while satisfying the maximum curvature and collision avoidance constraints [10]. Hu, H. et al., proposed a new algorithm called APF-D3QNPER, combining the action output method of APF (artificial potential field) with the dueling double deep *Q*-network algorithm and introduced experience sample rewards in the experience playback portion of the traditional DRL (deep reinforcement learning) algorithm, overcoming the limitations of traditional path planning methods in unknown environments, such as reliance on high-precision maps, lack of generalization ability, and obstacle avoidance capability [11]. Xie, R. et al., presented a DRL approach for three-dimensional path planning utilizing local information and relative distance without global information to make up for the deficiencies of traditional path-planning algorithms in a complex and dynamic environment [12]. The above AI-based path-planning methods can effectively support the path-finding tasks of drones in three-dimensional complex environments but ignore dynamic perception in complex environments.

Animals have excellent navigation abilities. Drawing inspiration from animal environmental perception and navigation mechanisms, bio-inspired perception, navigation, and path-planning technologies provide a possibility to further solve intelligent path-planning issues in complex and unknown environments [13,14]. The neuromorphic visual system event camera is regarded as a potential candidate for improving the rapidity of perception and path planning for drones in dynamic environments. Compared with traditional machine vision systems, biology-inspired event cameras have significant advantages over traditional cameras: low response delay, low data rate, high dynamic range, and low power consumption, providing new ideas for improving the performance of existing AI-based path-planning algorithms. Although there are currently some studies on event-based reinforcement learning, these methods are mainly applied to fields like traffic signals, intelligent trains, multi-agent systems, etc. [15,16,17]. However, there is little research using the airsim unreal platform to simulate real dynamic characteristics and physical features, such as collisions, for the event-driven reinforcement learning training of unmanned aerial vehicles. In this paper, an intelligent perception and path-planning method of drones based on event camera and reinforcement learning named E-DQN is proposed to address the drawbacks of traditional algorithms and make up for the lack of application of event-driven reinforcement learning algorithms in the field of drone decision-making in airsim. Environment perception employing event data can effectively improve the rapidity of path-planning systems. Reinforcement learning draws inspiration from the dopamine reward and punishment mechanism in the brain and has strong environment adaptability and flexibility, which makes it a potential candidate for solving path-planning problems in large-scale and dynamically changing environments. Deep *Q*-network (DQN) is a commonly used optimization algorithm for reinforcement learning, which employs a neural network to represent the optimal policy function *Q* and updates the parameters. Reinforcement learning training by DQN can generate optimal value functions through interaction with the environment without prior knowledge [18]. Introducing event streams into DQN for path planning can ensure the stability and efficiency of training, overcoming the shortcomings of traditional decision algorithms. The proposed algorithm was validated on the airsim simulation platform [19]. The experiment results show that the optimal path to the target can be found after several rounds of training.

The remainder of this paper is structured as follows: Section 2 describes the overall framework of the proposed path-planning algorithm. The detailed method is explained in Section 3. Section 4 presents simulation experiments developed on a virtual flight platform supported by airsim and unreal engine. Section 5 concludes the whole work and discusses future research.

## 2. System Framework

**Figure 1:**
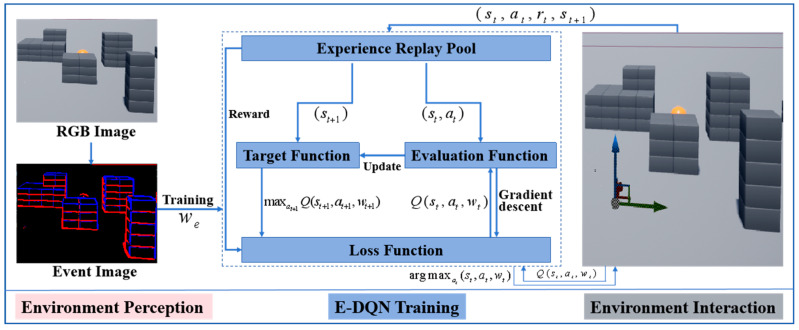
The overall framework of bio-inspired path planning system.

Path planning is performed to generate a motion strategy with which a drone can find an optimal path connecting the starting and ending positions. Considering the complex task execution environment of drones, a bio-inspired path-planning algorithm based on event perception and reinforcement learning was designed to perform fast decision-making for drones. The overall framework is as Figure 1.

The system framework consisted of three parts, including environment perception, E-DQN training, and environment interaction. Event data were employed in the bio-inspired path-planning system for perception, which greatly reduced data redundancy and improves the training efficiency of DQN [20]. Due to the asynchrony and sparsity of event data, an auto-encoder was introduced to process the event stream data and make them available for the following training network [21]. The DQN algorithm requires multiple episodes of training through interactions between drones and environments. Airsim serves as a plugin for any virtual environment provided by the unreal engine and is regarded as an ideal platform for artificial intelligence training [22]. Unreal engine simulator creates a highly realistic virtual environment with high fidelity and avoids situations where drones are prone to crashes while flying in the real environment [23]. In addition, airsim can equip drones with multiple sensors and provide rich API interfaces to support the task execution of drones in different scenarios.

## 3. E-DQN-Based Path-Planning Method

### 3.1. Bio-Inspired Environment Perception

Drones equipped with RGB cameras have reaction times of tens of milliseconds, which is not enough for drones to perform fast navigation tasks in complex dynamic environments. An event-based camera is a novel visual sensor inspired by animal perception mechanisms. The time resolution of the event camera is in the microsecond range. Compared with traditional cameras that capture images at a fixed frame rate, event cameras have the advantage of low latency, high dynamic range, low power consumption, and high time resolution. Moreover, events are inherently generated by changes in brightness typically arising from motion, which makes event cameras natural complex environment perception sensors and a good fit for fast path planning with obstacle avoidance function. Therefore, event data were employed for environmental perception in this paper.

Airsim is a simulator built on an unreal engine that supports the realistic physical and visual simulation of drones or cars. Airsim provides an event camera data collection simulator that allows drones to fly and collect event data in a virtual environment, which is equivalent to equipping a drone with an event camera. To prevent damage to drones caused by flying in real environments, the airsim virtual simulator is utilized for event data collection. Event cameras generate “events” by measuring logarithmic brightness changes. A set of events contains four values, namely pixel position (x and y coordinates), timestamp, and polarity. An event has a polarity of +1 or −1 based on an increase or decrease in logarithmic brightness. Event data in airsim are obtained using a series of transformations of RGB images. An event occurs when the absolute change in logarithmic brightness exceeds a certain threshold. Event sequences are reported in the form of byte streams, which construct an event accumulation on a 2D frame known as an “event image”. In event images, events with a polarity of +1 and −1 are visualized as red pixels and blue pixels, respectively.

To convert RGB images into event images, firstly, the logarithmic strength of the current frame was calculated [24].

(1) $$L(u,t)=\log\left(0.299I_{R}(u,t)+0.587I_{G}(u,t)+0.114I_{B}(u,t)\right),$$

where L(u,t) means the logarithmic strength of the current frame. $I_{R}(u,t)$, $I_{G}(u,t)$, and $I_{B}(u,t)$ represent the brightness of RGB images in the red, green, and blue channels, respectively. Subsequently, all pixels are traversed, and the polarity of each pixel is calculated based on the threshold of the difference in logarithmic intensity between the current frame and the previous frame. The polarity of the current frame can be obtained as follows:

(2) $$p(u,t)=\left\{\begin{array}{c} +1,\;if\;L(u,t)-L(u,t-1)>Th\\ -1,\;if\;L(u,t)-L(u,t-1)<-Th \end{array}\right.,$$

where Th denotes the event brightness threshold. According to the degree to which the intensity change exceeds the threshold, the number of events to be emitted for each pixel can be determined. Assuming that the maximum number of events that a pixel experience is $N_{\max}$, the total number of emissions simulated at the pixel position *u* can be expressed as follows:

(3) $$N_{\max}=\mathrm{int}\left(\frac{\Delta t}{r_{t}\times 10^{-3}}\right),$$

(4) $$N_{e}(u,t)=\min\left(N_{\max},\frac{\Delta L(u,t)}{TOL}\right),$$

where $r_{t}$ is the refractory period. $N_{e}(u,t)$ represents the number of events generated at pixel *u*. $N_{\max}$ represents the maximum constraint on the maximum number of possible events that can occur in a pixel. ΔL(u,t) is the total logarithmic brightness change that occurs at pixel *u* and time *t*.

To train the neural network architecture using event data, a one-dimensional event stream with event data form (*x*, *y*, *t*, *p*) was generated, where (*x*, *y*) denotes the sum coordinates of the current pixel, *t* is the timestamp, and *p* represents the polarity of the event. Then, the timestamp of each interpolation event was determined through interpolation, which represents the amount of time between the captured previous and current images.

(5) $$t=t_{prev}+\frac{\Delta T}{N_{e}(u)},$$

where $t_{prev}$ means the timestamp of the first event generated at the current time step. ΔT indicates the time interval between frames in microseconds. Finally, the generated event stream was sorted in timestamp order to simulate event data correctly.

### 3.2. Feature Extraction of Event Data

The generated event data cannot be applied to neural network-based motion decision algorithms directly because these data are not images but asynchronous event streams encoding changes in pixel intensity. The data processing of an asynchronous event stream is one of the key issues in utilizing event data for bio-inspired navigation and decision-making systems.

**Figure 2:**
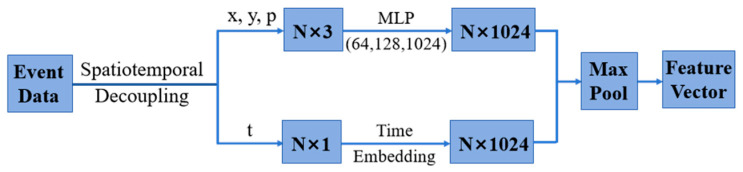
Spatiotemporal decoupling of event data.

With the resolution of the event camera set to (*H*, *W*), the event at time t was defined as a tuple (*t*, *x*, *y*, *p*). A series of events in the time window τ were represented as $E_{\tau }=\left\{e_{i}|t<i<t+\tau \right\}$. The events in $E_{\tau }$ can be accumulated and represented as the corresponding event image frames $IE_{\tau }$. Although event sequences are time-based data streams, the data length is long and difficult to apply to neural networks. Therefore, the decoupling of temporal and spatial information is needed to apply event data to path-planning and obstacle-avoidance algorithms. For spatial representation, pixel coordinates and polarity were encoded separately by calculating and extracting asynchronous event stream features. Moreover, the principle of position encoding was utilized for time embedding. For an event set $E_{n}$ with *n* events, the timestamp was normalized between 0 and 1 so that the timestamp corresponding to the end of the window was mapped to 1. Then, time characteristics were calculated for each normalized timestamp. The spatiotemporal decoupling process of event data is shown in Figure 2 [25].

**Figure 3:**
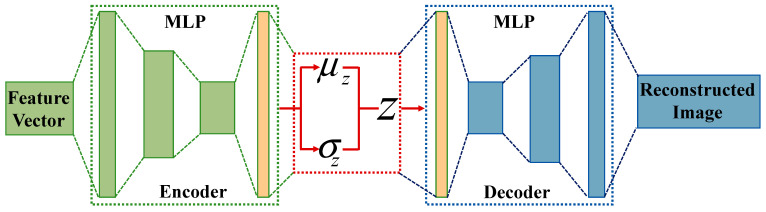
Auto-encoder of feature vector.

An auto-encoder is used to restore input data at the output and extract useful information from the data, which acts as a bridge for applying event data to reinforcement learning training. The auto-encoder describes a probabilistic framework for mapping feature vectors to reconstructed space rather than randomly mapping attributes to the output [26]. The auto-encoder attempts to learn parameter variable models by maximizing the marginal log-likelihood of training data composed of reconstruction loss and KL divergence loss, as shown in Figure 3.

Due to the narrow bottleneck layer in the middle of the network, only the most important information is preserved in the encoding value z. The decoder inputs a vector z and outputs a reconstructed image. Multi-layer perceptron (MLP) was used as a classification model for the encoder and the decoder. The activation function was implemented to solve the classification model of MLP. The commonly used activation functions include the sigmoid function, the tanh function, and the ReLU function. The sigmoid function and tanh function have a gradient saturation and output of non-zero mean at both ends of the function, which can affect the training effectiveness of the neural network. The ReLU function returns the input value when it is greater than 0 and 0 when it is less than or equal to 0 [27].

(6) ReLU(x)=max(0,x)

The ReLU function is simple and efficient in calculation without involving complex exponential operations. Moreover, the ReLU function can solve the gradient saturation problem of the sigmoid and tanh functions. Furthermore, the derivative of the activation function is always constant 1 in the positive region, which can effectively transfer gradients [28]. Therefore, the ReLU function was adopted as the activation function here. The training of the auto-encoder was performed end-to-end. The weights of the auto-encoder were generated simultaneously. The trained weights $w_{e}$ of the auto-encoder could be utilized as inputs for path-planning algorithms.

### 3.3. E-DQN Training of Drones

**Figure 4:**
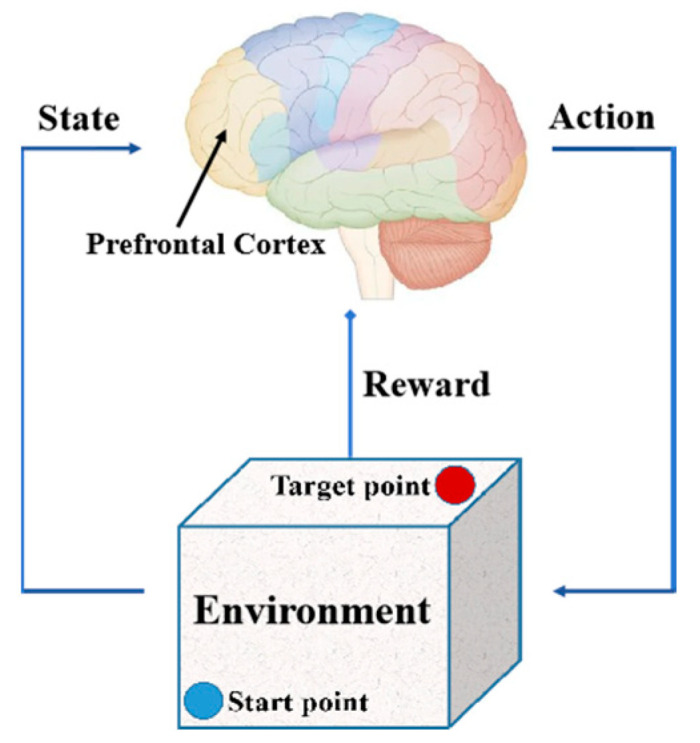
The decision-making mechanism of animals.

The path-planning problem refers to finding a collision-free path under the constraints of path length and training time. Reinforcement learning is a learning method inspired by animal dopamine reward strategies, suitable for solving path-planning problems through continuous trial and error. Animals produce movements through the prefrontal cortex. Dopamine is a neurotransmitter produced by the brain that can provide feedback on reward signals based on actions and states, thereby generating behaviors such as homing and foraging. The decision-making mechanism of animals is shown in Figure 4.

Inspired by an animal’s path-planning mechanism, reinforcement learning aims to learn motion policies for sequential decision problems by optimizing a cumulative future-reward signal. Reinforcement learning has exploratory characteristics and can continuously try new actions to discover reward signals during the learning process, which makes reinforcement learning highly adaptable and flexible when facing unknown environments or new tasks. Through exploration, drones can gradually learn the characteristics and laws of the environment, thereby generating optimal motion strategies. With event data representation value z as state observations, generating the next action by reinforcement learning can effectively solve the problem of the fast path planning of drones in complex environments.

*Q*-learning is one of the most popular reinforcement learning methods. However, in many cases, the state space of reinforcement learning tasks is continuous and infinite, making it difficult to store the value function in a table format [29]. The DQN algorithm can improve the processing ability of high-dimensional state spaces, providing a new idea to address the issues of rapidity and continuity in decision-making.

DQN approximates the action value $Q\left(s,a\right)$ by adopting a value function $Q\left(s,a;w\right)$, where w is the parameter for training the neural network [30]. Firstly, the original state $s_{t}$ and *Q* values of corresponding actions should be initialized. By providing starting position $O(O_{x},O_{y},O_{z})$, target position $T(T_{x},T_{y},T_{z})$ and event weights $W_{e}$, the next action selection of drones can be performed. In the decision-making process, ε−greedy strategy, which means a drone select actions randomly with a certain probability ε, is adopted to select an action and react with the environment to obtain a new state $s_{t+1}$ and reward *r*. The ε−greedy strategy helps to balance the trade-off between exploration and utilization, preventing getting stuck in a local optimum.

The training of path-planning neural networks is an optimization problem. The DQN algorithm includes two neural networks: the evaluated network (*Q*-value network) and the target network. The target network is to train the network by minimizing the mean square error of the *Q* value so that the network can approximate the real *Q* function. The objective function includes the difference between the actual reward and the target *Q* value of the next state. The calculation formula of the target network can be written as follows [31]:

(7) $$y_{t}=r+\gamma \cdot \max_{a}Q(s_{t+1},a;w,w_{e}),$$

where $y_{t}$ denotes the *Q* value of the target network, which represents the expected reward obtained after performing an action. *γ* describes the discount factor coefficient. $s_{t+1}$ denotes the next state. *a* is the selected action. *w* refers to the weights of the target network at time *t*. $w_{e}$ means event weights. $Q(s_{t+1},a;w,w_{e})$ represents the maximum *Q* value corresponding to the action in state $s_{t+1}$. *r* stands for the reward and can be written as follows:

(8) $$r=\left\{\begin{array}{l} 100,\;\mathrm{Reach}\;\mathrm{the}\;\mathrm{goal}\\ -100,\;\mathrm{If}\;\mathrm{collosion}\\ 0,\;\mathrm{Others} \end{array}\right.$$

To optimize the training network, the difference between the evaluated network and the target network is calculated as the following loss function [32]:

(9) $$L=1/2{\left[y_{t}-Q\left(s,a;w,w_{e}\right)\right]}^{2},$$

where *L* is the loss function. Then, the Q parameters of the neural network are updated using the back propagation gradient descent method to make the $Q(s_{t},a_{t})$ approach $y_{t}$. The updated *Q* network is the following [33]:

(10) $$Q=r+\left(\gamma *Q_{t+1}*(1-dones)\right),$$

where *dones* equals 0 or 1 indicates whether to complete a training session.

A transition of a quadruple $\left(s_{t},a_{t},r_{t},s_{t+1}\right)$ can be obtained through the update of the *Q* value. Then, a new state is entered to repeat the above process. After multiple training processes, the optimal path in the obstacle environment can be found. There is a strong correlation between adjacent transitions, which have an impact on *Q*-network training if transitions are used in order. Therefore, experience replay is introduced to eliminate correlation, provide convergence speed, and improve data utilization. A replay buffer that stores *n* transitions, known as experience, will be created. During each episode of training, batch-size transition data are selected randomly, and multiple random gradients are calculated to update the parameter *w* of the target *Q*-network. The flowchart of the proposed E-DQN training process for the bio-inspired path planning of drones in complex environments is shown in Algorithm 1.

**Algorithm 1.** E-DQN training process for bio-inspired path planning of drones in complex environments.1: Input: $\mathrm{Starting}\;\mathrm{point}\;O(O_{x},O_{y},O_{z})$$;\;\mathrm{Target}\;\mathrm{point}\;T(T_{x},T_{y},T_{z})$; Event weight *W_e_*2: Output: Optimal path3: Initialize: $\mathrm{Evaluation}\;\mathrm{function};\;\mathrm{Target}\;\mathrm{function};\;\mathrm{State}\;s_{t}$
4: $\mathrm{for}\;t<t_{end}$
5: $\mathrm{Select}\;\mathrm{an}\;\mathrm{action}\;a_{t}$ by the ε−greedy strategy.6: $\mathrm{Input}\;a_{t}$$\;\mathrm{into}\;\mathrm{the}\;\mathrm{environment}\;\mathrm{and}\;\mathrm{obtain}\;\mathrm{new}\;\mathrm{states}\;s_{t}$ and r.7:  $\mathrm{Compute}\;\mathrm{target}\;\mathrm{function}\;y_{t}=r_{t}+\gamma \cdot \max_{a}Q(s_{t+1},a;w)$
8:  $\mathrm{Compute}\;\mathrm{loss}\;\mathrm{function}\;L=1/2{\left[y_{t}-Q\left(s,a;w\right)\right]}^{2}$
9:  Introduce experience replay and update parameters 

$\;\;\;Q=r+\left(\gamma *Q_{t+1}*(1-dones)\right)$

10: **end for**

## 4. Experiments

Plan planning with obstacle avoidance means that drones can independently analyze the environment information and generate a collision-free path from the initial state to the target state in an environment with unknown obstacles. To verify the feasibility of the proposed algorithm, an obstacle-avoidance scenario built on the airsim platform was created.

### 4.1. Experimental Setup

**Figure 5:**
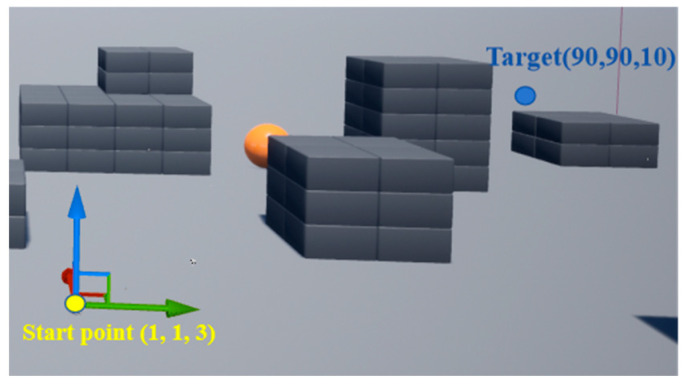
Flight environment.

The simulation was performed on a computer with a 2.40 GHz Intel Core i7 processor and an 8 GB GPU. To interact with airsim’s APIs, programming was implemented using the Python language. The flight range of the drone was a three-dimensional obstacle environment of 110 m × 110 m × 25 m, as shown in Figure 5.

In the airsim flight environment, the positions of the yellow dot and the blue dot were regarded as the starting point and the target point, respectively. The gray blocks and yellow ball were considered obstacles. The start position and the target position were set to (1, 1, 3) and (90, 90, 10), respectively. The goal of the experiment was to enable a drone to find an optimal and collision-free path from the start to the target. The path-planning method was trained using E-DQN. To ensure that the drone is well-trained, the total training timesteps were set to 5 × 10^5^. The main parameters utilized in the experiment are shown in Table 1.

### 4.2. Experimental Results

**Figure 6:**
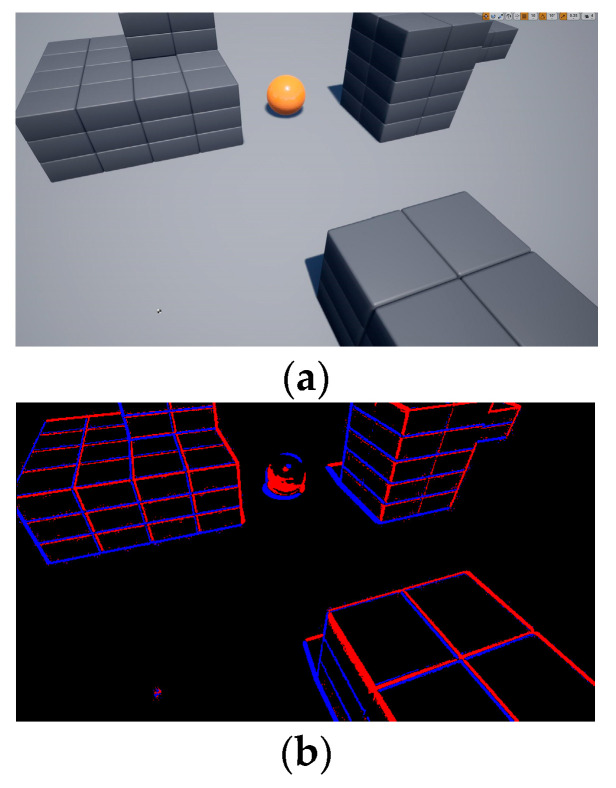
Comparison of RGB image and event image. (**a**) RGB image. (**b**) Event image.

To collect environmental information, a drone is allowed to fly freely in the airsim environment to collect RGB image data and generate event data by calculating the brightness changes in adjacent RGB images. The comparison of the RGB image and the event image is shown in Figure 6.

**Figure 7:**
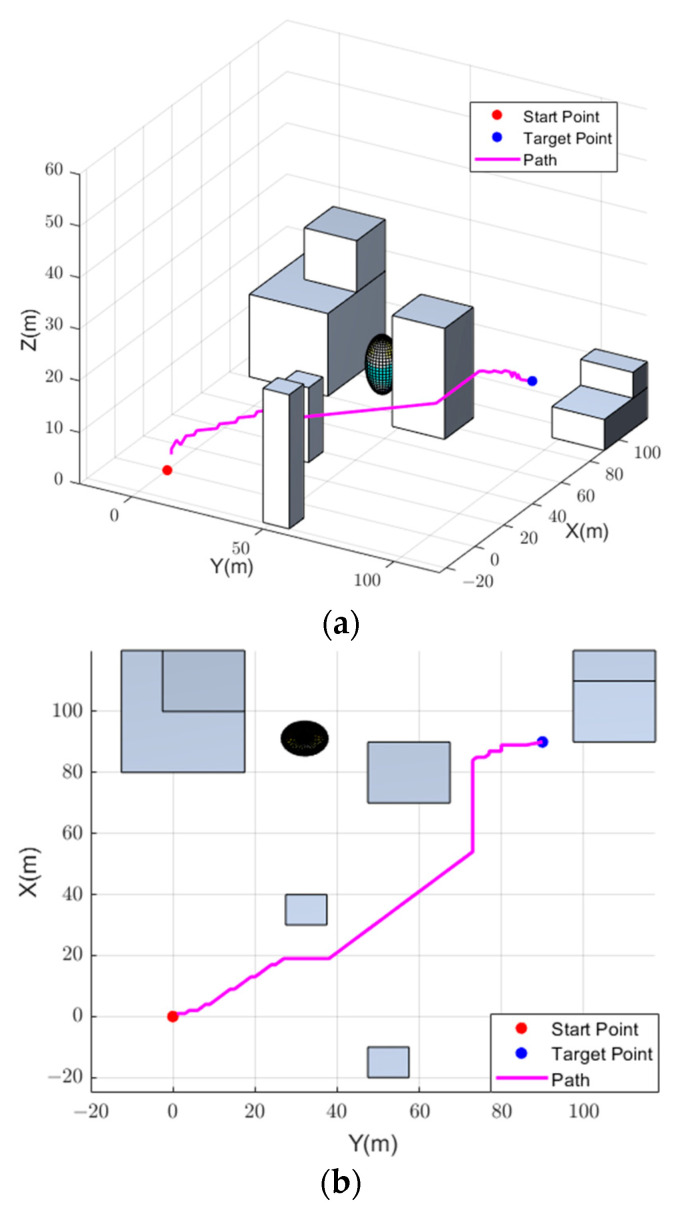
Planned path. (**a**) 3D View of the Planed Path. (**b**) Top View of the Planned Path.

Then, event weights generated by the auto-encoder were employed for DQN training and to obtain motion selection experience at a faster pace. Due to the lack of experience in the early stages of training, the drone randomly selected actions to explore the environment. The random exploration period was set as 1000. Moreover, the ε−greedy strategy was adopted to select the next action of drones during one training cycle. When the drone entered a termination state due to a crash, reaching the target point, or exceeding the maximum step size, it entered the next training cycle. After training, the drone was able to reach the target point safely, and the motion trajectory of the drone could be obtained. To have a clearer view of the drone’s flight situation, a proportional sketch of the airsim environment and the final path are shown in Figure 7.

It can be seen from Figure 7 that the drone can find a collision-free and shortest path to reach the target after training using the proposed method. The running time of the entire planning process was 41.364 s. Moreover, the entire planning process consisted of 188 steps.

**Figure 8:**
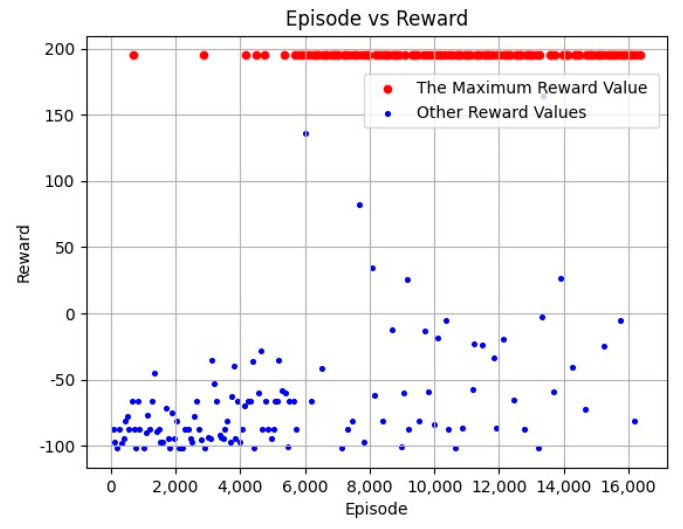
Episodes vs. Reward Result.

Figure 8 shows the reward values for different episodes. The total episodes was 16,381 during the training process. In Figure 8, the red dots represent the highest reward value of 195, while other reward values are represented by blue dots. It can be seen from Figure 8 that the training process converges gradually toward 6000 episodes, and most of the reward values can receive the maximum reward value during 6000–16,381 episodes, which proves the convergence speed of the proposed algorithm.

To further demonstrate the performance of the proposed method, the running time of the E-DQN path-planning method was compared with that of DQN and the spiking neural network (SNN) [31] in the same test environment. DQN is a commonly used intelligent path-planning algorithm, and SNN is also one of the typical brain-inspired path-planning methods. In our previous research, the running time of the SNN path-planning algorithm was introduced in detail [34]. The path-planning algorithm utilizing SNN adopts the leaky integrate-and-fire (LIF) model to generate a motion strategy. The parameter settings for SNN are shown in Table 2. The comparison results are shown in Table 3.

The path-planning time using the E-DQN algorithm was significantly reduced compared with that of the DQN algorithm and SNN algorithm, which verifies the rapidity and feasibility of the proposed algorithm.

## 5. Conclusions

To improve the rapidity of motion decision-making, a bio-inspired path-planning algorithm based on E-DQN for drones in complex environments was proposed. Unreal engine and airsim plugin were employed to build a training environment with obstacles for drones. Event data were generated using airsim simulator environment perception to reduce information redundancy. In terms of the asynchrony and sparsity of event data, an auto-encoder was designed to generate event weights, which were then input into a reinforcement learning network for training. Subsequently, DQN was introduced for the motion planning training of drones, and the ε-greedy strategy was used for drone action selection. The next step, the state of the drone, could be updated by setting reward signals and interacting with the airsim simulation environment. After several training sessions, the drone could find an optimal collision-free path to the target. The key contributions of this paper are listed below.

(1) An event auto-encoder for the unsupervised representation learning is presented from fast and asynchronous spatiotemporal event byte stream data.
(2) Motion policies are trained over event representations using the DQN for path planning for drones, which is superior to traditional path-planning algorithms.
(3) The dopamine reward mechanism is adopted for obstacle avoidance, which is more in line with animal action decision-making behavior.

Future work will investigate the extension of our algorithm to multiple drones and dynamically growing maps for motion decision-making and conduct real-world field experiments to validate our method. However, the real-world application of the proposed method also has limitations and challenges, such as efficiency issues, security issues, interpretability issues, and the practical challenges and scalability of the proposed method in diverse real-world scenarios. Overall, the problems of the E-DQN algorithm in practical applications are diverse and complex. However, with the continuous deepening of research and the continuous development of technology, many problems are expected to be solved or alleviated.

## Figures and Tables

**Table 1:** Parameter settings of DQN.

Parameter	Value
Discount rate	0.99
Time constant	0.005
Batch_size	128
Hidden_dim	16
Learning_rate	0.0004
Num_episodes	40,000
Max_eps_episode	10
threshold	200
State_dim	42
Action_dim	27

**Table 2:** The parameter settings for SNN.

Parameter	Value
membrane time constant	0.002
rest membrane potential	0 mV
membrane resistance	20 M
pre-amplitude	1.0
post-amplitude	1.0
pre-time constant	0.0168
post-time constant	0.0337

**Table 3:** Runtime comparison of typical brain-inspired path-planning methods.

Method	Run Time (s)
E-DQN	41.364
DQN	161.88
SNN	155.4

## Data Availability

The data presented in this study are available on request from the corresponding author due to the research project.
